# Towards an understanding of the roles of visual areas MT and MST in computing speed

**DOI:** 10.3389/fncom.2014.00092

**Published:** 2014-08-08

**Authors:** Andrew Isaac Meso, Claudio Simoncini

**Affiliations:** ^1^Institut de Neurosciences de la Timone, UMR 7289 CNRS and Aix-Marseille UniversitéMarseille, France; ^2^Department of Neurobiology, University of ChicagoChicago, IL, USA

**Keywords:** motion perception, ocular following, speed estimation, spatio-temporal frequencies, MT, MST

Estimating object motion within dynamic visual scenes is a critical skill for animals. In primates, this is supported by cortical neurons identified in Primary Visual Cortex (V1) and so called motion areas Middle Temporal (MT) and adjacent Medial Superior Temporal area (MST, which receives MT inputs) of macaques (Van Essen and Maunsell, 1983). Previous work has uncovered neural mechanisms serving motion direction estimation but there remain gaps in understanding the computation and representation of speed in the brain and the roles MT and MST play.

Recently published work (Miura et al., 2014) characterized the response properties of MT and MST neurons of the rhesus monkey during a brief presentation of moving grating stimuli. The large field stimulation evoked fast (~55 ms in monkey) reflexive ocular following responses (OFR; Miles et al., 1986) during which spiking rates of neurons in the two areas were measured. OFR is thought to rely on fast motion estimation computations which occur in part in MT and MST to stabilize gaze by driving the eyes at a speed and direction dependent on the stimulation (Kawano et al., 1994; for review Masson and Perrinet, 2012). Thus, OFR accurately reflects early activity through very few synaptic connections in the cortical motion pathway, making it an excellent behavioral probe of sensory processing.

Miura et al. (2014) recorded neural responses within a 60 ms window starting 20 ms before eye movements. Using a luminance grating stimuli they directly probed how MT sensitivity related to MST sensitivity before comparing both to behavioral responses. By recording spatiotemporal frequency responses in different cells during OFR, they elucidated speed estimation and representation mechanisms. Their paradigm exploits Fourier Theory, which states that any signal comprises a set of sinusoidal gratings added together each with different amplitudes. Thus, testing a range of discreet points of stimulus temporal and spatial frequency parameters defines experimentally targeted scales linking behavioral task performance to neural computation performed by units with specific spatial and temporal scales of sensitivity or receptive fields. This stimulus constrains the ecological interpretation as sine waves are not typically seen in natural scenes so the assumption of linearity critical in the Fourier approach must be considered when generalizing findings to more complex scenes. The authors fitted the discreetly sampled points of spatio-temporal responses of each MT and MST unit with a two dimensional function composed of spatial and temporal Gaussian terms. This allowed them to estimate the frequency response profile of each unit and use a regression of these to estimate the population response in each area. Interestingly MST is seen to have a broad spatial frequency sensitivity extending to lower frequencies where there is little or no sensitivity in MT. They suggest that MT extracts motion at finer scales while MST is responsible for coarser scales. However, both showed similar optimal temporal frequencies around 20 Hz. As both structures have parallel outputs to the Pontine Nucleus which drives OFR, this leads to the question: does the critical locus of this driving role depend on the scales of stimulation?

In the fitting estimating unit response profiles used by Miura et al. (2014), the temporal Gaussian contained a parameter Q that quantified the diagonal orientation of the response in frequency space. Diagonally oriented or *inseparable* responses (*Q* = 1) indicate speed sensitivity independent of spatial features. Conversely, profiles elongated across spatial frequencies showing a constant optimum temporal frequency are *separable* (Q≈0), characteristic of temporal frequency tuning. Units showed a continuum of Q values; MT had a median of 0.25 making it more speed tuned than MST at 0.07. The low values surprisingly imply that most units in these *motion* areas have speed sensitivity that depends on spatial features. A comparison of this MST result to V1 population Q values of 0.08 (Priebe et al., 2006) shows similar speed tuning in MST and generic visual area V1. Previous work using a different method to estimate separability found a higher proportion of MT units (~0.25) to be speed tuned (Perrone and Thiele, 2001). Separability estimates depend on how the sparsely sampled frequency space is fitted with a 2D function and the use of symmetric functions by Miura et al. influenced estimates (Perrone, 2006). The frequency bandwidth of units makes them sensitive not just to sine waves but to features like edges ubiquitous in natural scenes. Neural recordings over longer presentations (>700 ms compared to 60 ms) found the tuning of initially less separable units to be more separable under broadband stimulation (Perrone and Thiele, 2001; Priebe et al., 2003). Human psychophysics experiments with presentations times comparable to Miura et al. (2014) found that increasing frequency bandwidth strengthens OFR reducing variability, but degrades speed comparison judgments (Simoncini et al., 2012). While presentation time effects and task specificity remain unclear, speed estimation under natural stimulation seems to exploit the disambiguating effects of broadband frequencies (Priebe et al., 2006; Meso and Zanker, 2009).

Miura et al. (2014) filled a void in the literature by characterizing MST frequency responses and comparing that with MT to reveal differences in preferred spatial scales. They mapped out the OFR spatio-temporal frequency responses finding sensitivity similar to MST, but shifted toward higher frequencies. They showed that the OFR profile could be reconstructed by a linear summation of MT and MST profiles, suggesting both contribute through parallel and not serial computations to drive OFR. This raises the question: What might the roles of MT/MST be when OFR is replaced by or accompanied by perceptual judgments?

There remain open questions about characteristic *separability* and specific roles of both areas when processing natural scenes. Theoretical work continues to resolve how unit outputs from an MT under broadband stimulation might be transformed to resemble MST units and better predict behavior (Perrone, 2012). Human speed perception and action (OFR) seem to involve different information integration strategies (Simoncini et al., 2012). There is much work left to standardize methods for characterizing sparsely sampled frequency responses and compute separability, understand processing under naturalistic stimulation and probe the parallel pathways and their implications for perception and action computations.

## Conflict of interest statement

The authors declare that the research was conducted in the absence of any commercial or financial relationships that could be construed as a potential conflict of interest.
